# Australian Notes

**Published:** 1920-09-18

**Authors:** 


### AUSTRALIAN NOTES.

FROM A SPECIAL CORRESPONDENT.

### New Health Act.

The new Health Act for the State of Victoria was to have come into operation on March 1. Actually it was not proclaimed until the 25th inst. On that day the old much-derided "Board of Health" met for the last time, passed the minutes of the previous meeting, and locked the doors. Henceforward our public health will be a matter for the "Commission of Public Health" to deal with. This body consists of seven members—the Chief Health Officer (by virtue of his office as Chief Health Officer), Dr. E. Robertson (late Chairman of the old Board of Health), two other medical practitioners, one member representing Metropolitan municipalities, one member representing cities, towns, and boroughs other than Metropolitan municipalities, one member representing shires other than Metropolitan municipalities, and a sanitary engineer.

The Chief Health Officer shall be appointed and may be removed by the Governor in Council. The others shall be entitled to hold office for three years from the date of appointment (except in the case of a member appointed to fill a casual vacancy, when such a member shall be entitled to hold office only for the unexpired portion of the period for which his predecessor was appointed), but he will be eligible for reappointment.

Appointed members (not being officers of the public service) shall be paid—"(*b*) an attendance fee of two guineas for each meeting attended by them at which a quorum is present; but so that no such member shall receive more than one hundred and twenty pounds as attendance fees in respect of any one financial year. The quorum shall be not less than five."

All these fees and expenses, it is understood, are to be paid out of moneys provided by Parliament.

The Act evidently intends to map the whole State into areas, with a health officer in charge of each, but the Government will probably content itself with half-a-dozen or so to begin with.

